# Battey’s Operation in Germany

**Published:** 1880-01

**Authors:** 


Battey's Operation in Germany.—Dr. Schucking (Centralblatt fur Gynakologie, den 27 September, 1879), performed Battey's operation on a woman 37 years of age, the mother of six children. The grounds for the operation were marked hysteria, epileptic attacks, metrorrhagia, with painful menstruation and trismus. Bromide of potassium, iron, massage, baths, valerian, morphia, chloral hydrate, rubbing in tincture of iodine over the region of the avaries, and the administration of Flower's solution, were ineffectual. It was, therefore, resolved to operate. The ovaries were removed through an abdominal incision, and the pedicles secured with a catgut. The Listerian method was employed in all its details. The spray was 2½ per cent. solution of carbolic acid. Hardly a drop of pus was present in the wound on the tenth day after the operation. The recovery was rapid and the result of the operation most gratifying. The abdominal pain, the uterine bleeding, the cataleptic attacks, the trismus and other symptoms had vanished. The patient now feels well and healthy. Microscopical examination of the ovaries gave no positive results, scarcely any discernible pathological changes having taken place.—*London Medical Record.*

